# Is CT or MRI the optimal imaging investigation for the diagnosis of large vestibular aqueduct syndrome and large endolymphatic sac anomaly?

**DOI:** 10.1007/s00405-019-05279-x

**Published:** 2019-01-11

**Authors:** S. E. J. Connor, C. Dudau, I. Pai, M. Gaganasiou

**Affiliations:** 1 School of Biomedical Engineering and Imaging Sciences Clinical Academic Group, King’s College, London, UK; 2 Department of Radiology, Guy’s and St. Thomas’ Hospital, London, UK; 3 Department of Neuroradiology, Ruskin Wing, Kings College Hospital, Denmark Hill, London, SE5 9RS UK; 4 Department of Ear, Nose and Throat Surgery, Guy’s and St Thomas’ Hospital, London, UK; 5 251 General and VA Air Force Hospital, Athens, Greece

**Keywords:** Magnetic resonance imaging, Computed tomography, Large vestibular aqueduct syndrome, Large endolymphatic sac anomaly, Inner ear, Deafness

## Abstract

**Background and purpose:**

We explored whether there was a difference between measurements obtained with CT and MRI for the diagnosis of large vestibular aqueduct syndrome or large endolymphatic sac anomaly, and whether this influenced diagnosis on the basis of previously published threshold values (Valvassori and Cincinnati). We also investigated whether isolated dilated extra-osseous endolymphatic sac occurred on MRI. Secondary objectives were to compare inter-observer reproducibility for the measurements, and to investigate any mismatch between the diagnoses using the different criteria.

**Materials/methods:**

Subjects diagnosed with large vestibular aqueduct syndrome or large endolymphatic sac anomalies were retrospectively analysed. For subjects with both CT and MRI available (*n* = 58), two independent observers measured the midpoint and operculum widths. For subjects with MRI (± CT) available (*n* = 84), extra-osseous sac widths were also measured.

Results

There was no significant difference between the width measurements obtained with CT versus MRI. CT alone diagnosed large vestibular aqueduct syndrome or large endolymphatic sac anomalies in 2/58 (Valvassori) and 4/58 (Cincinnati), whilst MRI alone diagnosed them in 2/58 (Valvassori). There was 93% CT/MRI diagnostic agreement using both criteria. Only 1/84 demonstrated isolated extra-osseous endolymphatic sac dilatation. The MRI-based LVAS/LESA diagnosis was less dependent on which criteria were used. Midpoint measurements are more reproducible between observers and between CT/MR imaging modalities.

**Conclusion:**

Supplementing MRI with CT results in additional diagnoses using either criterion, however, there is no net increased diagnostic sensitivity for CT versus MRI when applying the Valvassori criteria. Isolated enlargement of the extra-osseous endolymphatic sac is rare.

## Introduction

MRI and CT are widely and variably used for the evaluation of potential inner ear developmental anomalies in patients with audio-vestibular symptoms. The most frequent macroscopic inner ear abnormality demonstrated on imaging studies is that of the large vestibular aqueduct (LVAS) as shown by CT [1–5] or large endolymphatic sac anomaly (LESA) as shown by MRI [6–8]. The LVAS/LESA syndrome is characterised by progressive, fluctuating, unilateral or asymmetric sensori-neural or mixed hearing loss [9, 10]. The diagnosis of LVAS/LESA is important to explain audiological findings, to allow genetic testing and counselling, to advise the patient about avoiding contact supports, to direct a search for additional labyrinthine anomalies on imaging and to potentially plan and assess risks (e.g. perilymphatic gusher) of cochlear implantation.

There remains controversy as to whether CT or MRI is more accurate for the diagnosis of this inner ear anomaly [11]. Although previous literature supports a superior diagnostic yield for CT, the only previous study to specifically address this issue was performed using older MRI technology [8] and a recent meta-analysis recognised a paucity of data from other smaller series [12–18]. Since MRI is now the principle imaging modality used in the evaluation of congenital or progressive sensorineural hearing loss (SNHL) and asymmetric cochlear thresholds, it is important to determine whether an additional CT is required to increase diagnostic sensitivity for LVAS.

There are potential reasons for a discrepancy between CT and MR diagnosis. First, there may be a difference between measurements obtained within the vestibular aqueduct (CT) or intra-osseous endolymphatic sac/duct (MRI) at the midpoint (mid post isthmic) and opercular segments. Threshold measurement values to distinguish LVAS are available for these locations on the basis of previous studies [4, 5, 19] but not for differing MRI sequences. Second, there may be cases where the extra-osseous endolymphatic sac is clearly dilated on MRI yet the intra-osseous components are within normal limits and hence there is no abnormality demonstrated on CT [12]. This situation may be more common in patients with short vestibular aqueducts. There are currently no recognised size criteria for an enlarged extra-osseous endolymphatic sac, although there is anatomical data indicating a range of normal size values [20, 21].

**Fig. 1:**
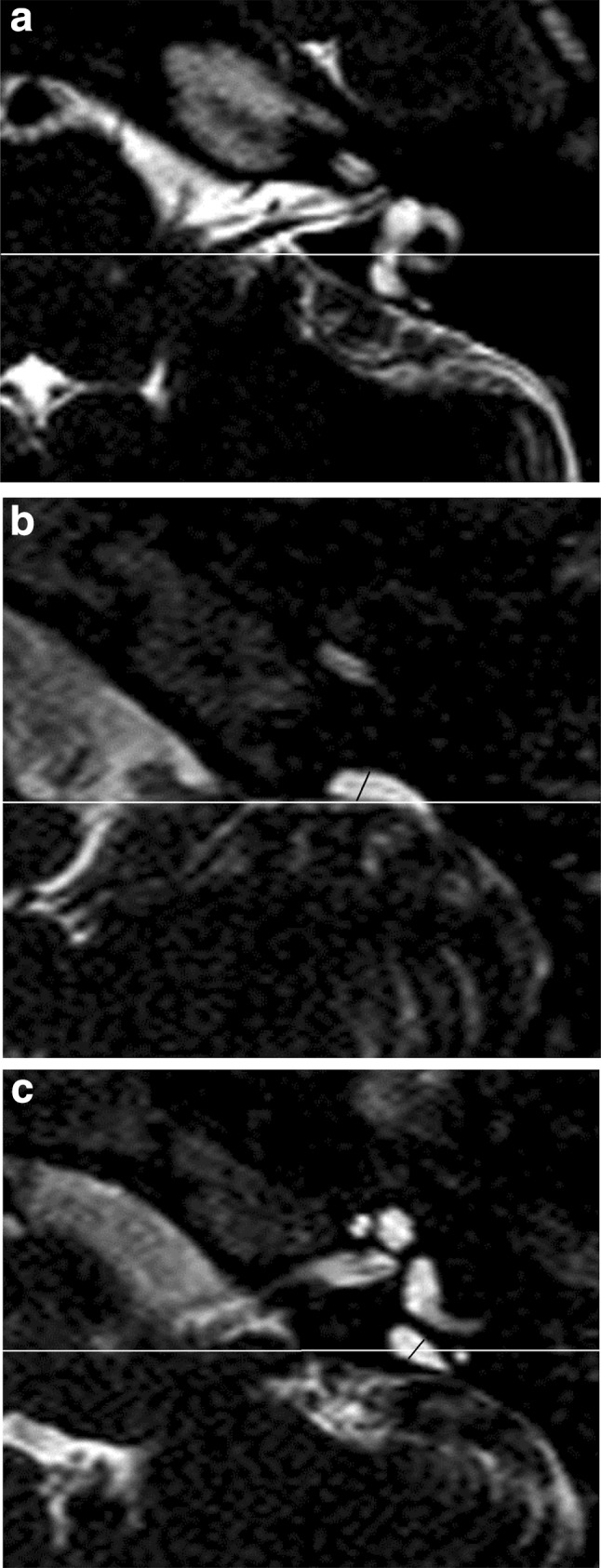
Intra-osseous measurement methodology. T2 DRIVE axial MR images demonstrating the vestibular, opercular and midpoint planes in a patient with bilateral large endolymphatic sac anomaly but no septations. **a** White line corresponds to the vestibular plane defined by the horizontal plane at the level of the dorsal common crus as it arises from the vestibule. **b** White line corresponds to the opercular plane defined by the horizontal plane at the level of the superior opercular lip. Black line corresponds to the opercular measurement. **c** White line corresponds to the midpoint plane, defined as halfway anteroposteriorly between the vestibular and opercular planes. Black line corresponds to the mid-point measurement

In addition to the imaging modality used, there may be variations in diagnostic yield as a result of inter-observer reproducibility and the use of differing measurement based diagnostic criteria. The criteria for diagnosing LVAS (CT) or LESA (MRI) are not absolutely standardised although two measurement criteria are more widely published and applied clinically [4, 5, 19]. First, the Valvassori criteria [19] indicate that the vestibular aqueduct is considered enlarged when > 1.5 mm at the midpoint (Fig. 1). Although initially applied to an oblique (Poschl) plane on hypocycloidal polytomography, this is now usually applied to axial sections. Second, the Cincinnati criteria refer to an enlarged vestibular aqueduct as a width ≥ 2 mm at the operculum (Fig. 1) and/or ≥ 1 mm at the midpoint (Fig. 1) on axial images [5].

We aimed to determine whether there was a difference between measurements obtained within the vestibular aqueduct (CT) or intra-osseous endolymphatic sac/duct (MRI) and whether this influenced diagnosis on the basis of previously published Valvassori and Cincinnati threshold values [5, 19]. We also explored whether the extra-osseous endolymphatic sac could be dilated on MRI yet the intra-osseous component be within normal limits, and whether there was a correlation between extra-osseous and intra-osseous measures. Secondary objectives were to compare inter-observer reproducibility for the CT and MRI based measurements, and to investigate any mismatch between the diagnoses when applying the Valvassori versus the Cincinnati measurement criteria.

## Materials and methods

The study underwent local institutional review and was considered to represent service evaluation without a requirement for informed consent.

Subjects diagnosed with LVAS/LESA were identified and recorded by two observers over a 8-year period from 2009 to 2016. The diagnosis of LVAS/LESA for the purpose of inclusion was established on the basis of either the Valvassori or Cincinnati criteria as measured on either CT or MRI, or by the subjective assessment of an enlarged extra-osseous sac on MRI. Subjects had been referred for temporal bone MR and CT imaging with a range of audio-vestibular symptoms. The inclusion in the study did not require the LVAS/LESA to be symptomatic.

A range of different MRI and CT scanners and scanning parameters were employed due to the prolonged study period. While MRI was performed on 1.5 T systems in all cases, a variety of thin section T2/T2*-w sequences were utilised with slice thickness ≤ 0.7 mm, with > 90% of MRI studies being performed in the same institution driven equilibrium radiofrequency reset pulse (DRIVE), CISS, sampling perfection with application-optimized contrasts using different flip angle evolutions (SPACE) and TSE 3D. CT was performed on ≥ 16 slice scanners, with > 90% of the CT studies being performed in the same institution on 16 or 40-slice CT scanners (Philips Brilliance 16 and 40, Philips Healthcare, the Netherlands) with parameters of mA 100, kV 120, field of view 180 mm, 0.348, slice thickness 0.67 mm, reconstruction index 0.33 mm and pixel spacing 0.23 mm (40 slice scanner) or 0.27 mm (16 slice scanner). Cases with slice collimation/thickness > 0.8 mm or with suboptimal imaging (motion artefacts, susceptibility artefacts from hearing devices) were excluded (*n* = 53).

There were 84 ears in 66 subjects (M/F: 26/40, age mean 22.7 years, range 2–64 year; 18 bilateral and 66 unilateral cases) with MRI available for retrospective review; and 58 ears in 53 subjects (M/F, 22/31 age mean 25 years range 2–61 years) with both MRI and CT available for retrospective review. Two independent observers (CD with 8-year radiology experience and MG with 7-year radiology experience) measured the midpoint and operculum vestibular aqueduct widths/short axes (CT), endolymphatic/duct intra-osseous widths/short axes (MRI) and extra-osseous endolymphatic sac widths/short axes (MRI). Readers were blinded to any other data and the CT and MR imaging was analysed in a random sequence by the observers.

CT and MRI digital data were reviewed on a GE Centricity PACS workstation (GE Medical Systems, Milwaukee, Wisconsin). MRI and CT studies of the individual patient were reviewed separately. Axial images only were assessed with standardised magnification. Images were viewed with a CT window width/centre of 4000/400, whilst MRI window settings were optimised to define the endolymphatic sac with the window width widened until a “penumbra” at the margins of the sac started to appear. The mean value for each parameter was subsequently used for analysis purposes (calculated to the nearest 0.1 mm).

The midpoint measurement required an initial delineation of axial sections corresponding to the vestibular plane (a horizontal plane at the level of the dorsal common crus as it arises from the vestibule) (Fig. 1a) and the opercular plane (a horizontal plane at the level of the superior opercular lip) (Fig. 1b). The midpoint plane was defined as halfway between the vestibular and opercular planes (Fig. 1c) [5, 22]. The midpoint measurement was made on this axial section at the widest width perpendicular to the line of the vestibular aqueduct trajectory (Fig. 1c). The operculum measurement was the maximum perpendicular vestibular aqueduct width at the level of the operculum (Fig. 1b). Cases were evaluated against the Valvassori criteria (width > 1.5 mm at the midpoint) [19] and the Cincinnati criteria (width ≥ 2 mm at the operculum and/or ≥ 1 mm at the midpoint) [5].

To validate the initial subjective evaluation of enlarged extra-osseous endolymphatic sacs on MRI, extra-osseous endolymphatic sac widths contralateral to the symptomatic side were also measured in 50 additional subjects with dedicated thin section T2-w MRI studies encompassing the temporal bone in patients without audio-vestibular symptoms. Extra-osseous endolymphatic sac widths/short axes (perpendicular to the petrous ridge) were < 0.5 mm in all cases, and were considerably smaller than any included in the study group.

The CT and MRI based mean midpoint and opercular measurements were correlated with Wilcoxon rank sum test. Percentage agreement (within 0.5 mm) between mean CT and MRI based measurements was also evaluated. Cases in which there was a mismatch in diagnosis of LVAS/LESA using the Valvassori and Cincinnati measurement criteria were identified. The percentage agreement of CT and MRI in terms diagnosis of LVAS/LESA using the Valvassori and Cincinnati measurement criteria was calculated and the diagnostic yield was compared with the Chi-square test.

For cases in which there was a mismatch in diagnosis of LVAS/LESA; either when comparing CT versus MRI or when applying Vavassori versus Cincinnati criteria, the medical charts were reviewed to determine whether the clinical presentation was in keeping with the diagnosis of LVAS/LESA.

Cases were recorded, in which the extra-osseous endolymphatic sac was clearly dilated in short axis on MRI, whilst the intra-osseous component was within normal limits. Statistic correlation of short axis extra-osseous sac size with intra-osseous measurements was performed using the Pearson’s correlation coefficient.

Inter-observer reproducibility for the CT and MRI measurements was evaluated with intra-class correlation coefficient. Percentage agreement of the observers in terms of CT and MRI diagnosis of LVAS/LESA using the two criteria was also assessed.

Statistically significant difference was considered to be *p* < 0.05. All analyses were performed with IBM SPSS statistics version 24.0.0.

## Results

There was no significant difference (*p* > 0.05, Wilcoxon) between the midpoint (*p* = 0.76) and operculum measurements (*p* = 0.82) obtained with CT versus MRI. There was a 95% agreement between the mean midpoint measurements obtained with CT and those obtained with MRI. However, the agreement figure slightly decreased (90%) for mean operculum width measurements.

**Fig. 2:**
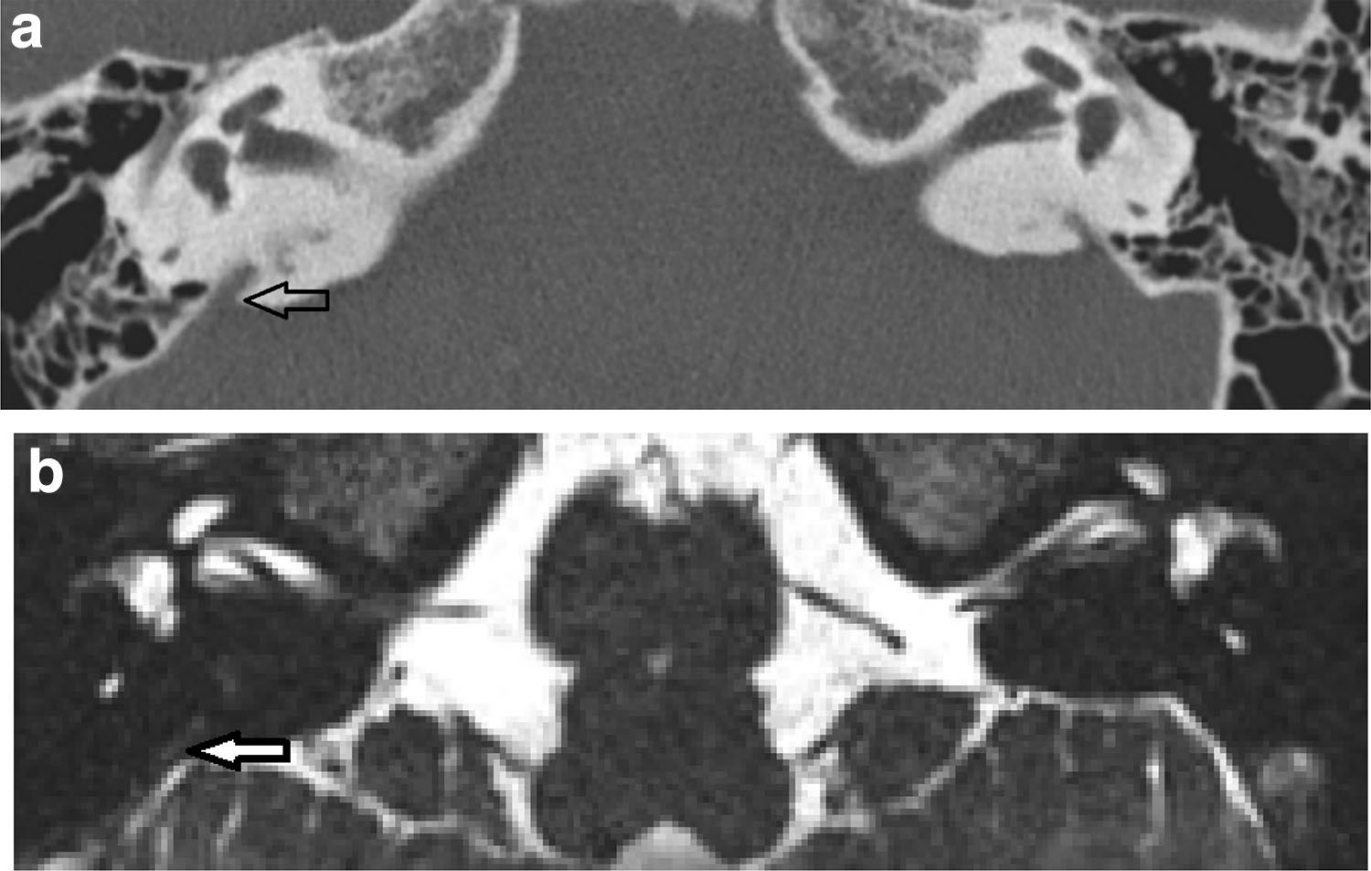
CT positive for LVAS but MRI negative for LESA. Axial CT (**a**) and T2 CISS axial MR image (**b**) demonstrates a case in which there was 2 mm measurement at the operculum on CT, thus Cincinnati criteria positive for LVAS (black open arrow) but not on MRI (white filled arrow)

CT diagnosed LVAS/LESA in 2/58 cases (Valvassori) and 4/58 cases (Cincinnati) when MRI measurements were normal (Fig. 2), whilst MRI diagnosed LVAS/LESA in 2/58 cases (Valvassori) and 0/58 cases (Cincinnati) where CT measurements were normal. There was a moderate to high clinical suspicion of LVAS/LESA in almost all cases where there was a discrepancy between CT and MRI, regardless of whether CT or MRI was positive (Table 1a). In discrepant cases, it was not clear that either imaging modality corresponded better with clinical diagnosis; however the most convincing correlation was in the two cases which were CT positive and MRI negative on Valvassori criteria. Overall, there was 93% CT/MRI diagnostic agreement and no significant difference was demonstrated between CT and MRI in terms of their ability to diagnose LVAS/LESA using either Valvassori or Cincinnati criteria (*p* > 0.05; Chi sq).

**Table 1:** The clinical status of the ears when there were discrepancies in LVAS/LESA diagnosis; either when comparing CT versus MRI (a) or when comparing Valvassori versus Cincinnatti criteria (b)

	Case number	Hearing loss	Clinical suspicion of LVAS/LESA	Imaging/clinical discrepancy on laterality
(a) CT v MRI discrepancies				
CT positive/MRI negative (Valvassori) *n* = 2	1	Progressive	High	None
	2	Progressive	High	None
CT positive/MRI negative (Cincinatti) *n* = 4	1	Acquired	Moderate	Hearing worse in contralateral ear without imaging abnormality
	2	Acquired/progressive	Moderate	Hearing worse in contralateral ear without imaging abnormality
	3	Acquired	Moderate	None
	4	Progressive	Low	None
MRI positive/CT negative (Valvassori) *n* = 2	1	Congenital	High	Hearing similar in contralateral ear without imaging abnormality
	2	Congenital/progressive	Moderate	Less marked hearing loss in contralateral ear without imaging abnormality
CT positive/MRI negative (Valvassori) *n* = 0	None			
(b) Valvassori versus Cincinatti criteria discrepancies				
Cincinatti positive and Valvassori negative *n* = 8				
CT only *n* = 4	1	Congenital	High	Hearing similar in contralateral ear without imaging abnormality
	2	Acquired	Moderate	None
	3	Congenital/progressive	Moderate	Less marked hearing loss in contralateral ear without imaging abnormality
	4	Progressive	Moderate	Less marked hearing loss in contralateral ear without imaging abnormality
CT and MRI *n* = 3	5	Congenital	High	None
	6	Congenital	High	None
	7	Progressive	Low	None
MRI only *n* = 1	8	Acquired	Moderate	None
Valvassori positive and Cincinatti negative *n* = 0	None			

**Fig. 3:**
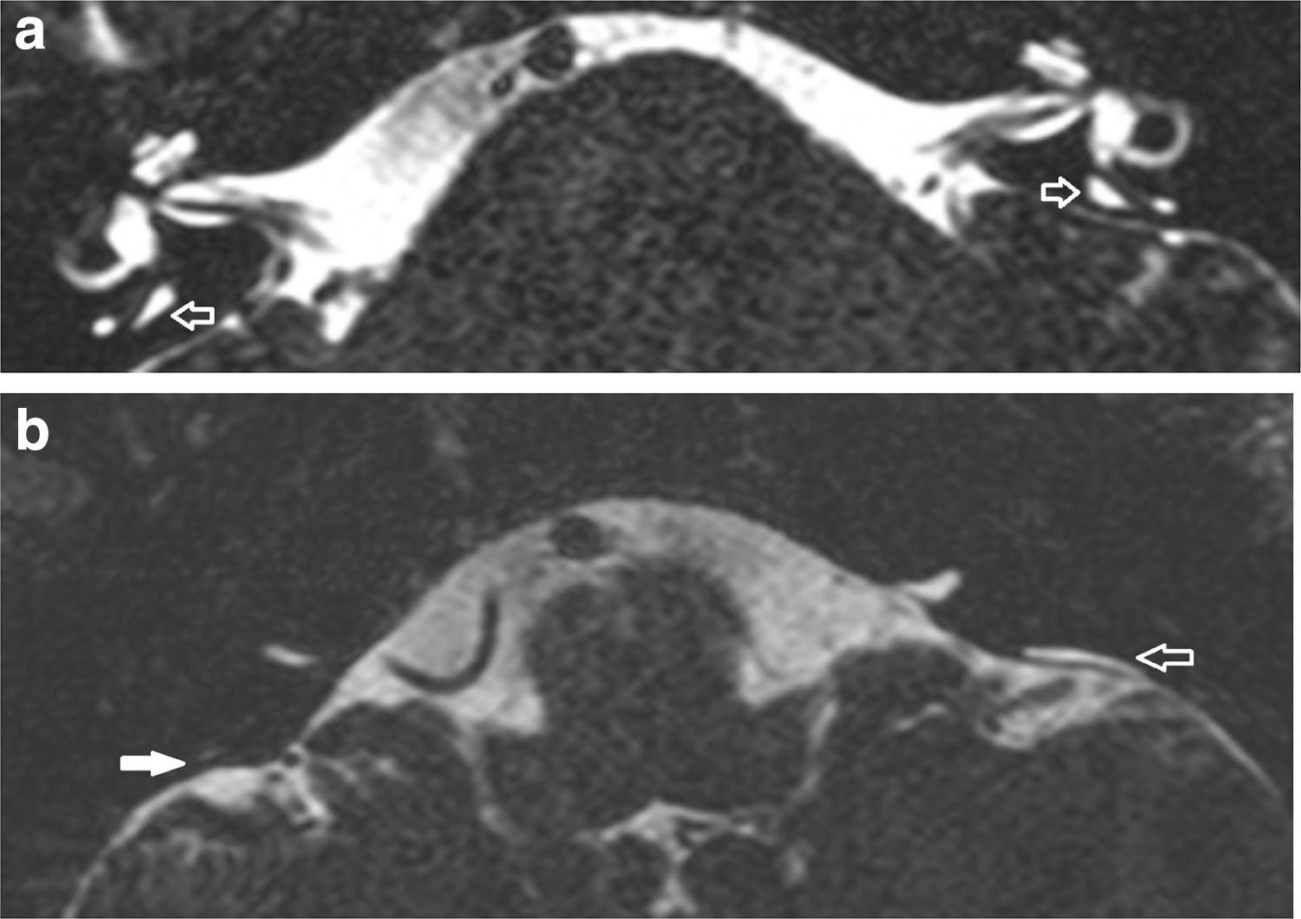
Intra-osseous measurements diagnose LESA on MRI but extra-osseous sac not enlarged. T2 DRIVE axial images show widened midpoint measurements bilaterally (white open arrows in **a**). There is an enlarged extra-osseous sac on the left (white open arrow in **b**) but not on the right (white filled arrow in **b**)

**Fig. 4:**
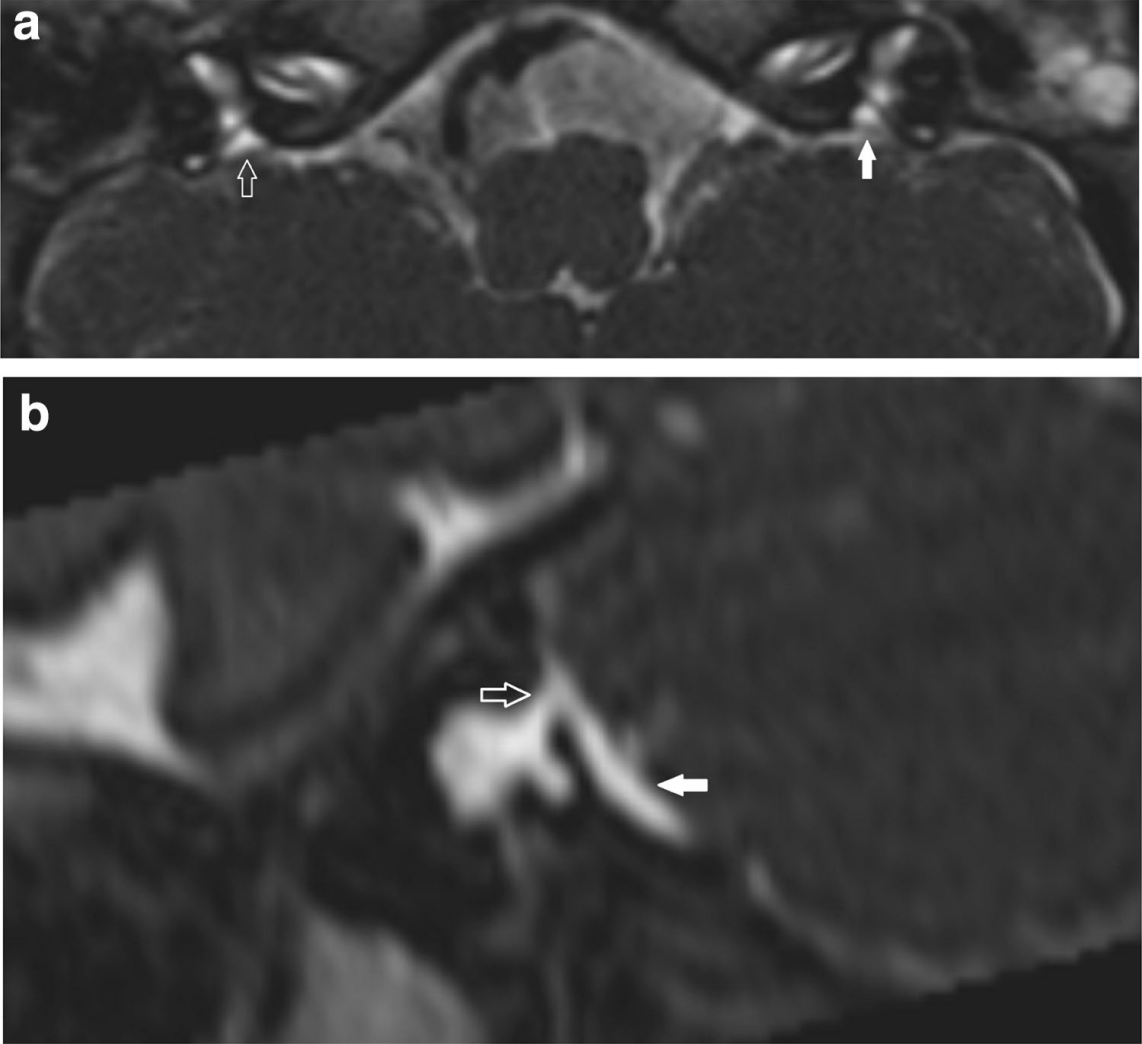
Isolated enlargement of the extra-osseous sac. T2 CISS axial image (**a**) demonstrates a very short splayed LESA without a clearly defined operculum and no widened intra-osseous measure is defined on axial images (white open arrow). An operculum is just defined on the left (white filled arrow). The sagittal oblique reformat **b** demonstrates a minimally prominent pre isthmic segment (white open arrow) but that the remaining LESA corresponds to an enlarged extra-osseous sac (white filled arrow)

We found enlargement of the extra-osseous endolymphatic sac to be frequently seen in cases with enlarged intra-osseous sacs/ducts on MRI, and the extra-osseous endolymphatic sac was only judged to be within normal limits in 8/84 cases (Fig. 3). In only one case was the extra-osseous endolymphatic sac clearly dilated on MRI whilst the intra-osseous component was within normal limits (Fig. 4). There was a weak but positive correlation between the extra-osseous endolymphatic sac width and the operculum measurement on MRI (*r*: 0.62). However, there was no correlation demonstrated between the extra-osseous endolymphatic sac width and the midpoint measurement.

**Fig. 5:**
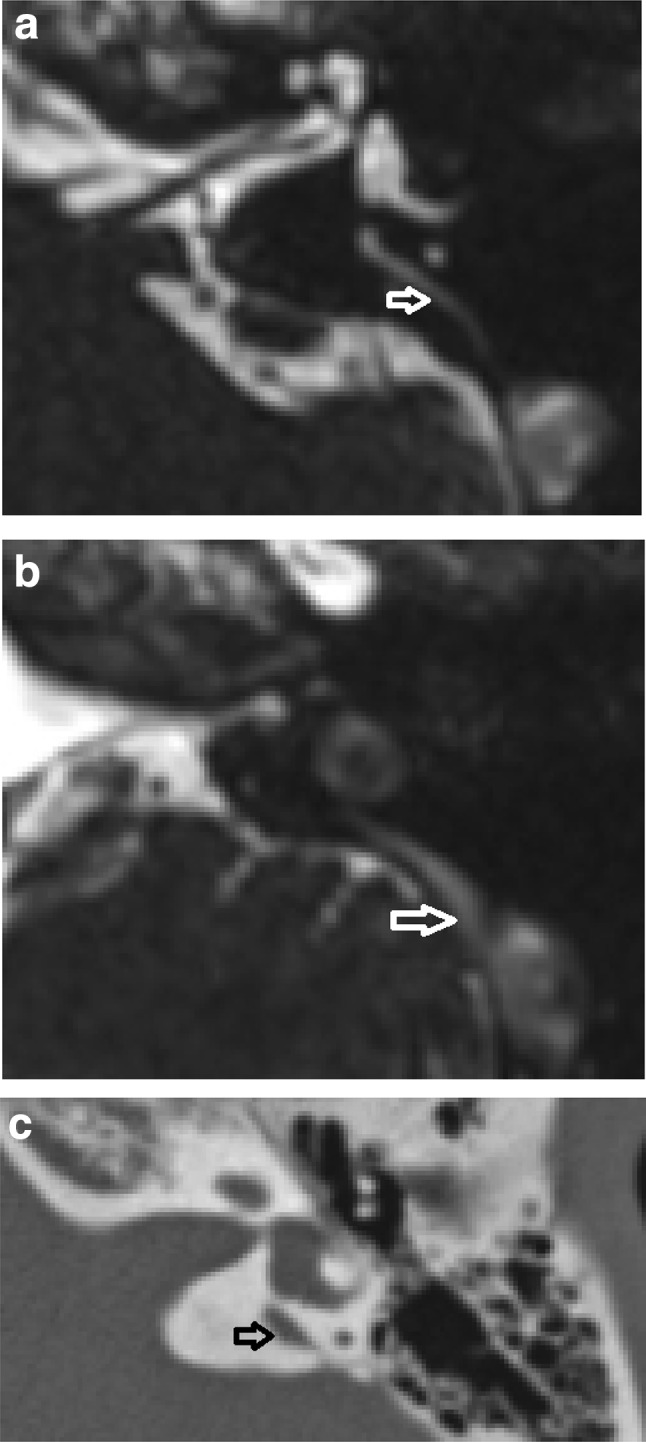
Cincinnati criteria positive but Valvassori criteria negative cases. T2 CISS axial images (**a**) and (**b**) demonstrate an elongated intra-osseous endolymphatic sac/duct. The midpoint (white open arrow in **a**) is not widened on either criteria. At the opercular portion, there is widening (white open arrow in **b**) so the case is Cincinnati criteria positive but Valvassori criteria negative. Note how it is difficult to define the transition between the bony operculum and the low signal dura overlying the extra-osseous sac. **c** A different patient demonstrated a minimally widened (1.2 mm) midpoint on CT so the case is also Cincinnati criteria positive but Valvassori criteria negative

There were 8 cases (4 on CT alone, 3 cases on both CT and MRI, 1 case on MRI alone) in which measurements indicated a LVAS/LESA diagnosis using the Cincinnati but not the Valvassori criteria (Fig. 5). Regarding these 8 cases where the mean measurement values diagnosed LVAS/LESA using the Cincinnati but not the Valvassori criteria, there was a mismatch in both observers measurements in 6/8 cases. Of note, the mismatches were due to the additional opercular criterion in 5/8 cases, the different midpoint criterion in 1/8 cases and both criteria in 2/8 cases. The clinical information was also reviewed for these cases (Table 1b). There was a moderate to high clinical suspicion of LVAS/LESA in those ears with diagnosis on CT alone, with 5/8 demonstrating a clear correspondence between the laterality of hearing loss and imaging abnormality. By definition, all Valvassori positive cases would also satisfy the Cincinnati criteria, thus there were no cases where measurements indicated a LVAS/LESA diagnosis using the Valvassori but not the Cincinnati criteria (Table 1b).

The inter-observer reproducibility was good to excellent for all CT/MRI based measurements, although optimal at the midpoint and with MRI based measurements. The inter-rater reliability values and percentage agreement values for all measurements are demonstrated in Table 2. The percentage agreement for the two observers in terms of CT and MRI diagnosis of LVAS/LESA using the Valvassori/Cincinnati criteria were 95/98% (CT) and 93/97% (MRI) respectively.

**Table 2:** IRR (inter-rater reliability) values for observations of each dimension

	IRR	% agreement
CT operculum	0.753	75
CT midpoint	0.892	89
MRI operculum	0.8	80
MRI midpoint	0.923	92
MRI short-axis extra-osseous sac	0.753	75

## Discussion

In broad terms, there are two indications for temporal bone imaging in the context of hearing loss: aetiological investigation and detection of aberrant anatomy relevant to surgical planning. Although CT was historically used to depict the inner ear structures, MRI is now generally accepted as the imaging investigation of choice for SNHL, and there is a trend to its primary use in the planning of cochlear implantation [18, 20, 21]. Previous literature has proposed that CT is the optimal imaging modality and is indicated for the diagnosis of a large vestibular aqueduct [11–18]. It is therefore important to establish whether CT provides any additional benefit for the diagnosis of LVAS/LESA as has been suggested by previous studies [12–18, 22]. Patients increasingly present with SNHL at a young age due to universal hearing screening programmes as well as increased access to audiology, and it is this paediatric group who are most sensitive to the effects of ionising radiation [23].

We have performed the largest comparison of CT and MRI data for the diagnosis of LVAS/LESA to date. The MRI-based intra-osseous measurements did not significantly differ from the CT based measurements and there was 93% overall agreement in terms of LVAS/LESA diagnosis. Any discrepancy may be due to differing interpretation of the site of measurement or differing depiction of the width measurement by CT and MRI. When comparing bony MRI and CT measurements on previous studies of the jaws and the cochlea, there has been shown to be no clear bias to either imaging modality, and with differences in comparable distances being < 1 mm [24, 25].

There were four cases in which CT diagnosed LVAS/LESA but MRI was normal, using the Cincinnati criteria, and this was due to the demonstration of increased opercular measures on CT compared with MRI. Percentage agreement between CT and MRI was also lowest at the operculum. It may be speculated that MRI-based measurements are reduced relative to those of CT at this site, due to the angulated bony structure of the operculum resulting in susceptibility and chemical shift effects. It may also reflect a difficulty in distinguishing the tip of the thin bony operculum from the adjacent low signal dura with MRI (e.g. Fig. 5b) and some heterogeneity in the signal returned by the complex structure of the endolymphatic sac. It has also been proposed that MRI measurements may be reduced relative to CT as a result of a previous transient enlargement of the fluid space that resulted in widening of the vestibular aqueduct [8].

The Valvassori criteria are entirely dependent on the midpoint measurement and, using these criteria, there were an equal number of LVAS/LESA cases diagnosed by MRI (*n* = 2) or CT (*n* = 2) alone. Potential causes of MRI measurements being increased relative to CT are a “blooming effect” of the fluid signal, partial volume effects of the increased voxel size, smoothing algorithms and the effect of our MRI standardised window level settings. There remains no standard of reference for the intra-osseous MRI measurements; however, a previous study reported midpoint diameters (mean 0.8 mm, range 0.5–1.4 mm) similar to those demonstrated with other imaging modalities [8] and it appears justified to compare with the established CT based criteria.

There was a moderate to high clinical suspicion of LVAS/LESA in almost all cases where there was a discrepancy between CT and MRI, regardless of whether CT or MRI was positive. Interestingly, the pattern and laterality of hearing loss was correlated best in those cases which were found to be CT positive and MRI negative on Valvassori criteria. Although it could be argued that only cases with equivocal enlargement are likely to result in discrepancies between CT and MRI diagnoses, it remains unclear as to whether there is a correlation between size and audiological findings [26–31], so such marginal cases may still be clinically significant.

We explored the role of an isolated enlarged extra-osseous endolymphatic sac may play in the imaging diagnosis of LESA. The extra-osseous sac is only demonstrated on MRI and hence has not featured in the pre-existing CT based criteria. There was only one case of isolated extra-osseous sac enlargement so it may be a limited addition to any MRI based diagnostic criteria. Moreover, the clinical significance of such isolated extra-osseous endolymphatic sac enlargement has not been explored. The clinical course in this ear was of congenital and progressive hearing loss. We found enlargement of the extra-osseous sac to be frequently seen (76/84) in cases with enlarged intra-osseous sacs/ducts on MRI, but unlike in previous reports, this was not present in all cases [8]. Our review of asymptomatic ears was in keeping with the finding of Dahlen et al. [8], with the extra-osseous endolymphatic sac not being clearly identified on MRI in most cases, and it always demonstrated a short axis < 0.5 mm. Microsurgical studies in normal subjects have shown highly variable width and height of 3.83 mm and 3.3 mm but the short axis is not reported [32].

Although there have been previous studies comparing CT with MRI for the diagnosis of LVAS/LESA, these are typified by small numbers of patients with concordant CT and MRI scans, older CT or MRI technology without information on imaging parameters, and limited details on imaging criteria used for diagnosis. In a dedicated study of LVAS/LESA imaging [11], there was an excellent correlation demonstrated between CT and MRI LVAS/LESA measurements; there were 5/38 ears in which CT alone demonstrated LVAS/LESA, whilst there was only 1/38 ears in which MRI alone diagnosed LVAS/LESA. Other authors have included imaging findings in children presenting with a range of hearing loss types and severity, of which some have recorded details of CT and MRI LVAS/LESA diagnoses [12–18]. In the three studies with larger numbers of LVAS/LESA diagnosed and both CT and MRI available, there were 26/26, 6/6, 10/10 patients diagnosed with CT and 0/6, 2/26 and 10/10 patients diagnosed with MRI [15, 16, 18]. It should be appreciated that there are some additional potential benefits of CT in terms of speed of scanning, and notably removing the need for anaesthesia and sedation in young patients. There is also improved of high spatial resolution definition of the facial nerve course and cochlear aperture in cochlear implant candidates; however, these advantages need to be weighed against the concerns about ionising radiation in individual cases.

The lack of standardisation of CT-based measurement thresholds introduces a further challenge to the consistency of LVAS/LESA diagnosis. Numerous upper limits to the vestibular aqueduct width ranging from 1 to 2 mm have been documented with microdissections, polytomographic studies and CT studies [8]. We evaluated the most widely applied contemporary CT-based criteria (Valvassori and Cincinnati criteria), and aimed to highlight any potential diagnostic discrepancy which may arise by applying different measurement criteria. This demonstrated a trend to increased diagnosis using the Cincinnati criteria; however, the difference was less pronounced when using MRI. Almost all the cases of Cincinnati positive but Valvassori negative ears had a moderate to high clinical suspicion and a pattern and laterality of hearing loss compatible with a diagnosis of LVAS/LESA. The additional opercular measurement appears to the principle reason for the mismatch between Cincinnati positive but Valvassori negative cases and is felt to be a valuable addition.

Our other secondary outcome was to investigate the reproducibility of the measurements. An acceptable inter-observer reproducibility is a key requirement for a measurement to be used for robust clinical diagnosis. A recent study has demonstrated CT-based dimensions of the vestibular aqueduct to be highly variable on axial and reformatted images [33]. Our data showed that optimal reproducibility was achieved using MRI-based measurements and particularly in relation to the midpoint measurement. A previous study of MRI base endolymphatic sac measurements also showed excellent reproducibility [29].

A potential area for criticism arises from the variable CT and MRI scanning parameters included in the dataset. This was as a result of the 8-year time period required to accumulate the appropriate number of cases from a single centre to perform a meaningful study, the incidence of LVAS being 1–1.3% in a large series of inner ear studies [9]. We set a minimum standard of collimation thickness to reduce the impact of heterogeneity in scanning technique; however, this resulted in a large number of excluded cases. The heterogeneous MRI scan parameters and non-standardised imaging planes may also have some benefits in terms of the wider relevance of the study outcomes to centres using a range of MRI scanners and sequences. In addition, we did not systematically review clinical data as this was frequently incomplete. However, we selectively reviewed cases in which there was a mismatch between diagnosis made, by either imaging modality (MRI v CT) or measurement criteria (Valvassori versus Cincinnati) to establish the clinical significance in these borderline cases. Finally, it could be argued that the diagnostic yield of MR and CT should be analysed in patients without a pre-existing diagnosis of LVAS/LESA, however, our approach was designed to maximise the detection of any differences in diagnosis between the two imaging modalities.

In conclusion, there is no significant difference between the CT and MRI based midpoint and operculum width measurements with a 93% agreement in terms of LVAS/LESA diagnosis for both criteria. There is, however, potential for additional diagnoses of LVAS/LESA to be made when MRI is supplemented with CT using either measurement criteria. This should be weighed up against the risk of ionising radiation, especially in young children, unless there is other indication for CT such as anticipation of significant abnormal temporal bone anatomy which may be important in surgical planning. Furthermore, CT does not demonstrate an overall increased diagnostic sensitivity when applying the Valvassori criteria, although those additional cases diagnosed with CT do correlate well with clinical findings. Isolated enlargement of the extra-osseous endolymphatic sac is rarely seen on MRI and hence is a largely theoretical benefit of MRI. The MRI-based LVAS/LESA diagnosis was less dependent on which measurement criteria were used, and it should be noted that midpoint measurements are more reproducible between observers and between CT/MR imaging modalities. The increased sensitivity for the diagnosis of LVAS/LESA using Cincinnati criteria is principally due to the inclusion of the opercular width criterion, and this further measurement appears to identify clinically compatible additional cases of LVAS/LESA.

## Abbreviations

LVAS: Large vestibular aqueduct syndrome
LESA: Large endolymphatic sac anomaly

## References

[1] Emmett JR (1985). The large vestibular aqueduct syndrome. Am J Otol.

[2] Jackler RK, De La Cruz A (1989). The large vestibular aqueduct syndrome. Laryngoscope.

[3] Urman SM, Talbot JM (1990). Otic capsule dysplasia: clinical and CT findings. Radiographics.

[4] Juliano AF, Ting EY, Mingkwansook V, Hamberg LM, Curtin HD (2016). AJNR Am J Neuroradiol.

[5] Vijayasekaran S, Halstead MJ, Boston M, Meinzen-Derr J, Bardo DME, Greinwald J, Benton C (2007). When is the vestibular aqueduct enlarged? A statistical analysis of the normative distribution of vestibular aqueduct size. AJNR Am J Neuroradiol.

[6] Brogan MA, Chakeres DW, Schmalbrock P (1991). High-resolution 3DFT MR imaging of the endolymphatic duct and soft tissues of the otic capsule. AJNR Am J Neuroradiol.

[7] Casselman JW, Kuhweide R, Deimling M (1993). Constructive interference in steady state 3DFT MR imaging of the inner ear and cerebellopontine angle. AJNR Am J Neuroradiol.

[8] Dahlen R, Harnsberger HR, Gray SD, Shelton C, Allen R, Parkin JL, Scalzo D (1997). Overlapping thin-section fast spin-echo MR of the large vestibular aqueduct syndrome. AJNR Am J Neuroradiol.

[9] Campbell AP, Adunka O, Zhou B, Qaqish BF, Buchman CA (2011). Large vestibular aqueduct syndrome: anatomic and functional parameters. Laryngoscope.

[10] Gropen Q, Zhou G, Whittemore K, Kenna M (2011). Enlarged vestibular aqueduct-review of controversial aspects. Laryngoscope.

[11] Deep NL, Hoxworth JM, Barrs DM (2016). What is the best imaging modality for diagnosing a large vestibular aqueduct?. Laryngoscope.

[12] Kachniarz B, Chen JX, Gilani S, Shin JJ (2015). Diagnostic yield of MRI for pediatric hearing loss: a systematic review. Otolaryngol Head Neck Surg.

[13] Ghogomu N, Umansky A, Lieu JEC (2014). Epidemiology of unilateral sensorineural hearing loss with universal newborn hearing screening. Laryngoscope.

[14] Haffey T, Fowler N, Anne S (2013). Evaluation of unilateral sensorineural hearing loss in the pediatric patient. Int J Pediatr Otorhinolaryngol.

[15] Parry DA, Booth T, Roland PS (2005). Advantages of magnetic resonance imaging over computed tomography in preoperative evaluation of pediatric coclear implant candidates. Otol Neurotol.

[16] Simons JP, Mandell DL, Arjmand EM (2006). Computed tomography and magnetic resonance imaging in pediatric unilateral and asymmetric sensorineural hearing loss. Arch Otolaryngol Head Neck Surg.

[17] Tarshish Y, Leschinski A, Kenna M (2013). Pediatric sudden sensorineural hearing loss: diagnosed causes and response to intervention. Int J Pediatr Otorhinolaryngol.

[18] Trimble K, Blaser S, James AL, Papsin BC (2007). Computed tomography and/or magnetic resonance imaging before pediatric cochlear implantation? Developing an investigative strategy. Otol Neurotol.

[19] Valvassori GE, Clemis JD (1978). The large vestibular aqueduct syndrome. Laryngoscope.

[20] Digge P, Solanki R, Vishwakarma R, Kumar S (2016). Imaging modality of choice for pre-operative cochlear imaging: HRCT vs MRI temporal bone. J Clin Diagn Res.

[21] Joshi VM, Navlekar SK, Ravi Kishore G, Reddy KJ, Kumar ECV (2012). CT and MR Imaging of the inner ear and brain in children with congenital sensorineural hearing loss. Radiographics.

[22] Hricak H, Brenner DJ, Adelstein SJ (2011). Managing radiation use in medical imaging: a multifaceted challenge. Radiology.

[23] Pearce MS, Salotti JA, McHugh K (2012). Radiation exposure from CT scans in childhood and subsequent risk of leukaemia and brain tumours: a retrospective cohort study. Lancet.

[24] Nael CJO, Pretterlieber M, Gahleitner A, Czerny C, Breitenseher M, Imhof H (1999). Osteometry of the mandible performed using dental MR imaging. Am J Neuroradiol AJNR.

[25] Connor SEJ, Bell DJ, O’Gorman R, Fitzgerald-O’Connor A (2009). CT and MR Imaging cochlear distance measurements may predict cochlear implant length required for a 360° Insertion. AJNR Am J Neuroradiol.

[26] Koesling S, Rsainski C, Amaya B (2006). Imaging and clinical findings in large endolymphatic duct and sac syndrome. Eur J Radiol.

[27] Naganawa S, Ito T, Iwayama E (1999). MR imaging of the cochlear modiolus: area measurement in healthy subjects and in patients with a large endolymphatic duct and sac. Radiology.

[28] Connor SEJ, Siddiqui A, O’Gorman R, Tysome JR, Lee A, Jiang D, Fitzgerald O’Connor A (2012). Large endolymphatic sac compartments and associated magnetic resonance imaging features: a study of their audiological and clinical correlates. JLO.

[29] Boston M, Halsted M, Meinzen-Derr J, Bean J, Vijayasakaren S, Arjmand E (2007). The large vestibular aqueduct: a new definition based on audiologic and computed tomography correlation. Otolaryngol Head Neck Surg.

[30] Ahadizadeh E, Ascha M, Manzoor N, Gupta A, Semaan M, Megerian C, Otteson T (2017). Hearing loss in enlarged vestibular aqueduct and incomplete partition type II. Am J Otolaryngol.

[31] Ascha MS, Manzoor N, Gupta A, Semaan M, Megerian C, Otteson TD (2017). Vestibular aqueduct midpoint width and hearing loss in patients with an enlarged vestibular aqueduct. JAMA Otolaryngol Head Neck Surg.

[32] Ammirati M, Aldo S, Feghali J (1995). The endolymphatic sac: microsurgical topographic anatomy. Neurosurgery.

[33] Quan Y, Gao XJ, Liu J (2018). Variability of vestibular aqueduct measurements among axial, single-oblique and double-oblique computed tomography images. J Laryngol Otol.

